# Molecular Basis of Bacterial Longevity

**DOI:** 10.1128/mBio.01726-17

**Published:** 2017-11-28

**Authors:** Kieran B. Pechter, Liang Yin, Yasuhiro Oda, Larry Gallagher, Jianming Yang, Colin Manoil, Caroline S. Harwood

**Affiliations:** a Department of Microbiology, University of Washington, Seattle, Washington, USA; b Department of Genome Sciences, University of Washington, Seattle, Washington, USA; c Key Lab of Applied Mycology, College of Life Sciences, Qingdao Agricultural University, Qingdao, Shandong Province, People’s Republic of China; Georgia Institute of Technology

**Keywords:** *Rhodopseudomonas palustris*, Tn-seq, growth arrest, starvation, stationary phase

## Abstract

It is well known that many bacteria can survive in a growth-arrested state for long periods of time, on the order of months or even years, without forming dormant structures like spores or cysts. How is such longevity possible? What is the molecular basis of such longevity? Here we used the Gram-negative phototrophic alphaproteobacterium *Rhodopseudomonas palustris* to identify molecular determinants of bacterial longevity. *R. palustris* maintained viability for over a month after growth arrest due to nutrient depletion when it was provided with light as a source of energy. In transposon sequencing (Tn-seq) experiments, we identified 117 genes that were required for long-term viability of nongrowing *R. palustris* cells. Genes in this longevity gene set are annotated to play roles in a number of cellular processes, including DNA repair, tRNA modification, and the fidelity of protein synthesis. These genes are critically important only when cells are not growing. Three genes annotated to affect translation or posttranslational modifications were validated as *bona fide* longevity genes by mutagenesis and complementation experiments. These genes and others in the longevity gene set are broadly conserved in bacteria. This raises the possibility that it will be possible to define a core set of longevity genes common to many bacterial species.

## IMPORTANCE

Bacteria in nature and during infections often exist in a nongrowing quiescent state. However, it has been difficult to define experimentally the molecular characteristics of this crucial element of the bacterial life cycle because bacteria that are not growing tend to die under laboratory conditions. Here we present and validate the phototrophic bacterium *Rhodopseudomonas palustris* as a model system for identification of genes required for the longevity of nongrowing bacteria. Growth-arrested *R. palustris* maintained almost full viability for weeks using light as an energy source. Such cells were subjected to large-scale mutagenesis to identify genes required for this striking longevity trait. The results define conserved determinants of survival under nongrowing conditions and create a foundation for more extensive studies to elucidate general molecular mechanisms of bacterial longevity.

## INTRODUCTION

Microbiological research has focused primarily on understanding the physiology and dynamics of bacterial cells and populations during rapid growth. However, this rapid growth state is likely highly unusual in nature. It is known that many bacteria, including many pathogens, enter a growth-arrested state in which they remain viable for considerable periods of time (1–3). Good examples of this are *Mycobacterium tuberculosis* (4–6) and biofilm-forming organisms such as *Pseudomonas aeruginosa* (7–9). Growth arrest can be caused by nutrient or energy limitation or by other factors. While the physiology of fast-growing bacteria is well characterized, relatively little is understood about how cells maintain life under nongrowing conditions.

The two main established models of nongrowing bacteria are the dormant spore state, exemplified by Gram-positive *Bacillus* spp. and *Clostridium* spp. (10–12), and starvation-induced growth arrest in Gram-negative bacteria (6, 13). Nongrowing cultures of Gram-negative bacteria are typically established by supplying a resource such as carbon, nitrogen, or oxygen in a growth-limiting amount (6, 13). Studies of such cultures have informed us about general strategies that bacteria use to persist in a growth-arrested state, including scavenging nutrients, using endogenous storage compounds like glycogen, or degrading cellular components such as lipids to maintain viability (13, 14). In addition, some species have been shown to survive growth arrest by rerouting metabolic pathways to make energy generation more efficient (15–17). Many of these strategies are deployed to meet the imperative of maintaining an electrochemical gradient across the cell membrane, a requirement for viability (18). Growth arrest due to any sort of nutrient limitation will eventually impact the ability of a bacterium to generate the energy needed for this critical function. Because of the importance of having an energy supply to maintain viability, many species of bacteria exhibit a large decrease in viability following an initial period of growth arrest. This has been well documented for *Escherichia coli*, for which a small fraction of a growth-arrested population will survive after an initial die-off in a cycle of growth and death by feeding on the nutrients released from dead cells (1, 13, 14, 19). This failure of most cells to survive long-term starvation as a homogenous population may explain why *E. coli* has not served as a good model for discovery of bacterial longevity genes.

**FIG 1:**
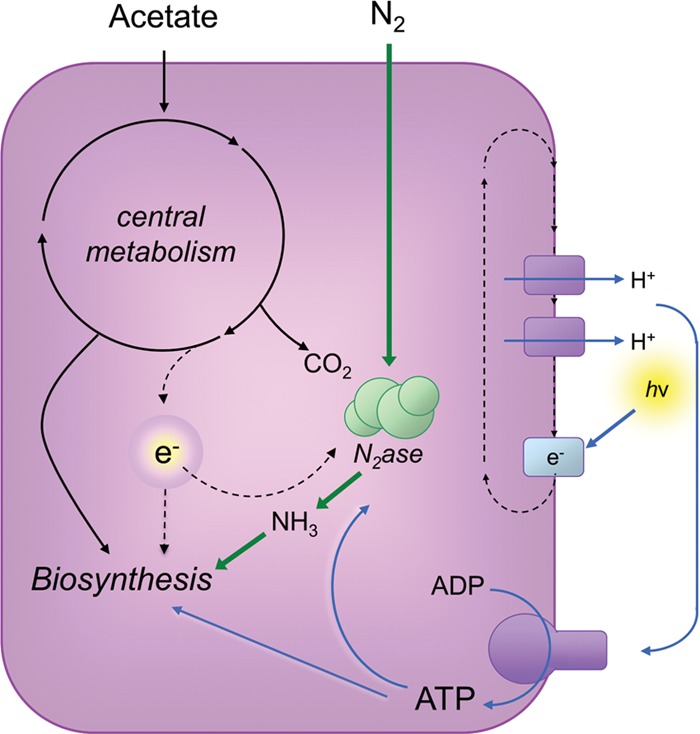
*R. palustris* metabolism. Cells for the experiments described in this article were grown anaerobically with light as the sole energy source and with acetate as the sole carbon source. Nitrogen gas (N_2_) or ammonia (NH_3_) was supplied as the nitrogen source. Under these conditions, a proton gradient generated by cyclic photophosphorylation provides energy for ATP synthesis. Acetate is used to support the production of cell biomass, and some electrons are diverted from acetate metabolism to nitrogenase (N_2_ase) for use in fixing N_2_ to NH_3_. In addition, some CO_2_ is released during acetate metabolism. In this work, cells were supplied with a growth-limiting amount of acetate, such that cells entered a growth-arrested state when all available acetate was exhausted. When in this state, *R. palustris* cells continue to generate ATP from light.

Several features of the physiology of the phototrophic alphaproteobacterium *Rhodopseudomonas palustris* suggest that it represents an ideal model for characterizing the molecular basis of bacterial survival in a nongrowing state. This bacterium can be put into a state of growth arrest due to nutrient limitation but not energy limitation (15, 20, 21). This would help remove from consideration genes that are important for cells to marshal resources to maintain an electrochemical gradient or to generate ATP to maintain viability. *R. palustris* generates ATP by cyclic photophosphorylation, and no portion of the carbon source that it is provided for biomass generation is used for energy (Fig. 1). Thus, *R. palustris* can enter into a growth-arrested state by carbon or nitrogen restriction, and cells remain viable for months when incubated in light (15, 20, 21). Here, we used transposon sequencing (Tn-seq) to identify genes required for growth-arrested *R. palustris* cells to remain viable. In complementary experiments, we analyzed the transcriptome of growth-arrested *R. palustris*. Over 100 genes were identified as essential for nongrowing *R. palustris* cells to maintain viability. Many members of this longevity gene set are conserved among bacteria, suggesting that common molecular mechanisms may support the longevity of diverse bacterial species.

## RESULTS

### Establishment of growth-arrested *R. palustris* cell populations.

**FIG 2:**
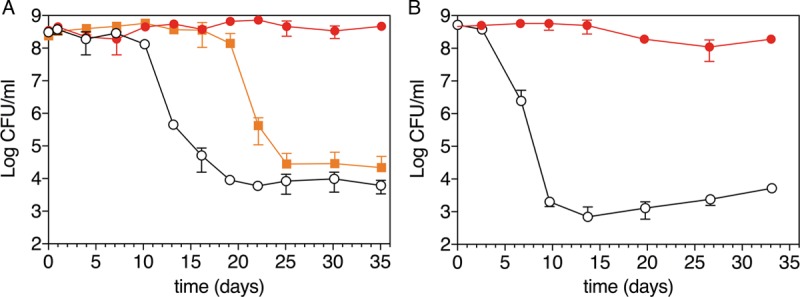
Viability of *R. palustris* after growth arrest. Growth arrest was initiated by two methods: (A) carbon limitation with N_2_ gas provided in excess as a nitrogen source or (B) carbon limitation with ammonium provided in excess as the nitrogen source. Growth-arrested cells were incubated anaerobically under constant light (red closed circles) or dark (black open circles) conditions. In panel A, cells were also exposed to 24 h of light following growth arrest and then placed in the dark for the remainder of the experiments (orange closed squares). Viability was measured by plating cells and counting CFU. The averages from two independent experiments are shown for each panel.

We grew cultures of *R. palustris* strain CGA009 anaerobically under conditions in which carbon but not energy was limited (Fig. 1). Under these conditions, nitrogen (N_2_ gas) and other resources, including energy from light, were provided in excess, while the carbon source, acetate, was provided in a growth-limiting amount. When acetate exhaustion resulted in growth arrest, we removed a subset of the cultures from light and incubated them in the dark for the duration of the experiment. The cultures that remained exposed to light remained fully viable over a period of 35 days, whereas the viability of dark cultures dropped by 4 orders of magnitude by approximately 10 days after the cells entered a growth-arrested state (Fig. 2). The results suggest that ATP generation through cyclic photophosphorylation is needed for long-term viability. However, these are population-level observations, and it is possible that the growth-arrested cultures that were incubated in light were continuously dying and growing in a cycle of balanced growth rather than maintaining continuous viability. To address this possibility, we examined growth-arrested cells by phase microscopy and after live/dead staining with propidium iodide. Propidium iodide permeates the membranes of dead, but not living, cells to stain DNA. Growth-arrested cells incubated in light were phase dense and had the same morphology as live cells, and we saw very few dead cells as assessed by propidium iodide staining. Cells incubated in the dark also tended to maintain their morphology, but many of the cells were less phase dense. Also, qualitative observations revealed that most dark-incubated cells stained red with propidium iodide after 10 days of growth arrest.

To investigate how the source of nitrogen impacts growth-arrested *R. palustris* survival, we carried out the same set of experiments with *R. palustris* cultures with 10 mM ammonia added. This concentration of ammonia represses the use of N_2_ gas as a nitrogen source by *R. palustris* (22). Growth-arrested cells provided with light as an energy source and ammonia as the nitrogen source (Fig. 2B) lost viability compared to cells that were given N_2_ gas as a nitrogen source (Fig. 2A); however, there was at most a 1-order-of-magnitude difference in the drop in viability between the two conditions over the 35-day experiments.

### The transcriptional response of *R. palustris* to growth arrest.

We observed that if we incubated growth-arrested cultures in light for 24 h beyond the initiation of growth arrest and then covered the tubes with foil and incubated them in the dark, the cultures maintained stable viable counts for approximately 10 days longer than cultures that were immediately placed in the dark (Fig. 2A). We wondered if the ability of *R. palustris* to survive in the dark for an extended period after a relatively brief light treatment might depend on the initiation of a light-dependent protective transcriptional response that occurs when growth ceases.

**FIG 3:**
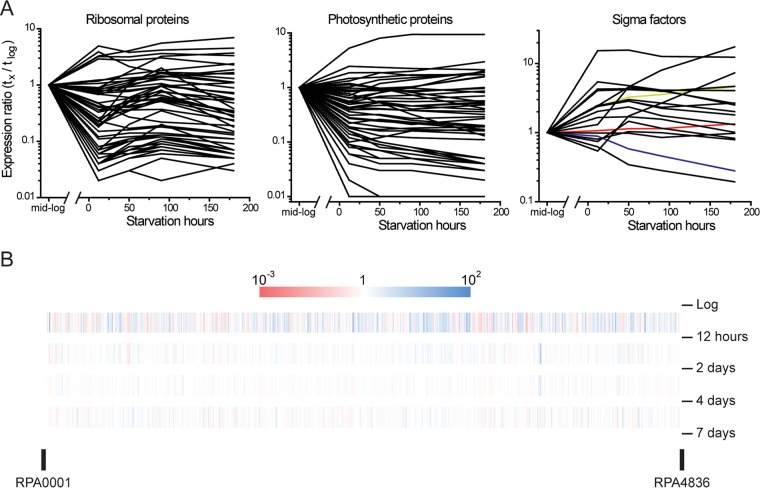
Transcriptional response of *R. palustris* to growth arrest. Cells were subjected to growth arrest by carbon limitation as described in the legend to Fig. 1A. (A) Expression levels of genes encoding ribosomal proteins, photosynthesis proteins, and sigma factors over time following growth arrest are shown. All numbers are normalized to each gene’s expression level during logarithmic growth. *R. palustris* encodes 19 RNA polymerase sigma factors. The expression levels of *rpoH* (*RPA0367*), *rpoD* (*RPA1288*), and *rpoN* (*RPA0050*) are shown in green, red, and blue, respectively. (B) Heat map of genome-wide expression level changes between the intervals. The numbers on the right are the intervals at which the samples were taken.

To address this, we extracted RNA from actively growing cells and from cells that were incubated in light for periods from 12 h to 180 h (0.5 to 7.5 days) after growth arrest and carried out transcriptome sequencing (RNA-seq). We found that cells responded rapidly to carbon starvation with most of the transcriptional responses occurring during the first 12 h after growth arrest. As seen in a previous study in which we looked at nitrogen starvation (15), genes encoding major functions that are normally highly expressed and needed for growth were turned down in expression (see Table S1 in the supplemental material). Included in this category are photosynthesis genes (*RPA1505* to -*1554*), genes for ribosomal proteins, and genes encoding ATP synthase (Fig. 3A). There are 4,836 genes in the *R. palustris* genome (23). By the seventh day after growth arrest, the expression levels of 257 genes were more than 10-fold lower than actively growing cells. Another 120 genes increased more than 10-fold in expression during this interval (Fig. 3B; Table S1). These include genes for many of the 19 RNA polymerase sigma factor genes carried by *R. palustris* (Fig. 3A) and genes involved in nutrient acquisition, including many of the genes required for anaerobic benzoate and 4-hydroxybenzoate degradation (*RPA0653* to -*0673*) and the *phn* gene cluster for phosphonate uptake and utilization (*RPA0689* to -*0702*), for example (Table S1).

10.1128/mBio.01726-17.3 TABLE S1  Transcriptome of *R. palustris* CGA009 at intervals following growth arrest due to carbon starvation. Download TABLE S1, XLSX file, 1.5 MB. Copyright © 2017 Pechter et al. 2017 Pechter et al. This content is distributed under the terms of the Creative Commons Attribution 4.0 International license.

10.1128/mBio.01726-17.4 TABLE S2  Tn-seq results. Fitness of *R. palustris* CGA009 genes at intervals after growth arrest due to carbon starvation. Download TABLE S2, CSV file, 1.8 MB. Copyright © 2017 Pechter et al. 2017 Pechter et al. This content is distributed under the terms of the Creative Commons Attribution 4.0 International license.

### Tn-seq screen to identify genes required for longevity.

Although many genes were elevated in expression following growth arrest, the approach of RNA-seq does not tell us if any of these genes are required for longevity. To identify longevity genes, we used Tn-seq, a high-throughput approach that takes advantage of next-generation sequencing to assess fitness defects and benefits conferred by transposon (Tn) insertion mutants within a saturating mutant pool. For this, we used a pool of *R. palustris* strain CGA009 transposon mutants described previously (24, 25) that consists of over 175,000 unique transposon insertion mutants. We inoculated the mutant pool into mineral medium containing a growth-limiting amount of acetate under continuous light illumination (as in Fig. 2A) and analyzed the effect of growth cessation on the makeup of the mutant pool. We sampled the cultures at the initiation of growth arrest and at 3, 7, 14, and 30 days post-growth arrest. We conducted two independent biological replicates of the experiment and processed the samples separately as described in Materials and Methods and previously (24). For each time point, genes with statistically significant (*P* < 0.05) read values below the mean in both replicates were noted. Cells with insertions in these genes, called reduced insertion fitness genes, were those present at a low frequency in the overall Tn pool. Genes with statistically significant values above the mean in both replicates were also identified. Mutants with Tn insertions in these genes were deemed to have increased insertion fitness. Genes with reduced and increased insertion fitness were recorded at the earliest experimental time point at which they met these criteria (see Tables S3 and S4 in the supplemental material). Genes that were previously classified as essential (*P* < 0.001) or that had reduced insertion fitness (*P* < 0.05) during aerobic growth in rich medium, or during anaerobic phototrophic growth in minimal medium, were excluded from our analysis (24, 25). Using this approach, we identified 117 genes that had reduced insertion fitness after 3 to 14 days of growth arrest (Table S3), comprising a longevity gene set.

10.1128/mBio.01726-17.5 TABLE S3  *R. palustris* longevity genes: genes with reduced fitness 3 to 14 days after growth arrest. Download TABLE S3, XLSX file, 0.1 MB. Copyright © 2017 Pechter et al. 2017 Pechter et al. This content is distributed under the terms of the Creative Commons Attribution 4.0 International license.

10.1128/mBio.01726-17.6 TABLE S4  Genes with increased insertion fitness 3, 7, 14, and 30 days after growth arrest. Download TABLE S4, XLSX file, 0.1 MB. Copyright © 2017 Pechter et al. 2017 Pechter et al. This content is distributed under the terms of the Creative Commons Attribution 4.0 International license.

### Categories of longevity genes.

More than one-third of the longevity genes (43 out of 117) were hypothetical genes or genes that did not fit into a known functional category (Table 1; see Table S5 in the supplemental material) (23, 26, 27). We also identified 34 genes belonging to the general category of biosynthesis and metabolism in the longevity gene set. These genes are involved in diverse functions, including nitrogen fixation, cofactor synthesis, and lipid synthesis. We expect that some of the genes in this category would differ depending on the conditions used prior to growth arrest and on the growth-limiting nutrient used to establish growth arrest. For example, we would not expect nitrogen fixation genes in the longevity gene set were we to carry out the Tn-seq experiment with cells grown with ammonia rather than nitrogen gas as the nitrogen source. A functional group that was overrepresented in the longevity gene set was transporters and transport-related proteins. Fifteen longevity genes were found in this category, 11 of which were ATP-binding cassette (ABC) transporters, a class of transporters that has high affinity for its substrates. This was more than 12% of the longevity gene set, twice overrepresented compared to 293 transporters or transporter-related genes among all *R. palustris* loci (6%). As would be expected from extensive studies with *E. coli*, *Bacillus subtilis*, and other bacteria, the RelA-SpoT homologue (RSH) *RPA2693* (annotated as *relA*) was found in our longevity gene set. RSH proteins are bifunctional enzymes that can synthesize and degrade the stringent response messenger (p)ppGpp (28, 29). (p)ppGpp regulates bacterial transcription globally in a number of ways in response to nutrient downshift (30–32). In addition, 10 genes classified as being involved in translation or posttranslational modification were identified as essential for *R. palustris* longevity in our Tn-seq screen. We verified the longevity functions of three of these genes in follow-up experiments described below.

10.1128/mBio.01726-17.7 TABLE S5  Functional categories of *R. palustris* longevity genes. Download TABLE S5, XLSX file, 0.04 MB. Copyright © 2017 Pechter et al. 2017 Pechter et al. This content is distributed under the terms of the Creative Commons Attribution 4.0 International license.

**TABLE 1:** Functional categories of genes essential for *R. palustris* longevity

Functional group	No. of genes (*n =* 117)	Functions of selected longevity genes
Biosynthesis and metabolism	34	Nitrogenase cofactor biosynthesis protein (NifB), alkaline phosphatase, hydrogenase/expression formation proteins, acetyl-CoA hydrolase
Hypothetical or unknown functional category	43	
DNA replication and repair	2	Smr protein/MutS2; Holliday junction resolvase-like protein
Cell wall/membrane biogenesis	2	Alanine racemase; UDP-*N*-acetylmuramoyl-l-alanyl-d-glutamate synthetase (MurD)
Signal transduction	3	Two-component sensor kinase (FixL), sensor signal transduction kinase of unknown function
Transcription	8	GTP-pyrophosphokinase (RelA), cold shock DNA-binding domain-containing protein (CspA)
Translation and posttranslational modification	10	tRNA uridine 5-carboxymethylaminomethyl modification protein (GidA), GTP-dependent nucleic acid-binding protein (EngD)
Transport	15	Twin-arginine translocation protein (TatB), high-affinity leucine-isoleucine-valine transport system periplasmic binding protein

### Tn mutants that restore hydrogen gas uptake in *R. palustris* strain CGA009 were positively selected after growth arrest.

In addition to identifying genes with reduced insertion fitness during growth arrest, we also identified 46 genes with increased insertion fitness following growth arrest (Table S4). Among the most readily explicable of these was a regulatory gene that indirectly represses expression of the NiFe uptake hydrogenase encoded by *R. palustris*. We previously determined that a deletion mutation of *RPA0980* (*hoxJ*) can override a regulatory mutation in our parent strain’s hydrogen uptake system such that cells acquire the ability to use H_2_ gas as an electron donor for carbon dioxide fixation (33). In our Tn-seq experiments, RPA0980::T24 insertion mutants outperformed all other mutants in the pool, and at 30 days post-growth arrest, they comprised 89 and 94% of the normalized reads in the two replicates, respectively. It is likely that these mutants used CO_2_ that accumulated in cultures when they oxidized acetate prior to growth arrest and H_2_ produced during nitrogen fixation to form biomass (see Fig. S1 in the supplemental material). At some point after day 14 of growth arrest, RPA0980::T24 insertion mutants outcompeted other Tn mutants sufficiently to start taking over the cultures.

10.1128/mBio.01726-17.1 FIG S1  Probable metabolic route to growth for RPA0980::T24 insertion mutants that were positively selected when cells were starved for carbon (acetate) under nitrogen-fixing conditions. These mutants allow a functional uptake hydrogenase enzyme to be expressed. CO_2_ produced from acetate prior to growth arrest and H_2_ produced along with ammonia as a product of nitrogenase can be used by the Calvin cycle to generate cell biomass. Download FIG S1, TIF file, 2.1 MB. Copyright © 2017 Pechter et al. 2017 Pechter et al. This content is distributed under the terms of the Creative Commons Attribution 4.0 International license.

### Validation of selected longevity genes.

**FIG 4:**
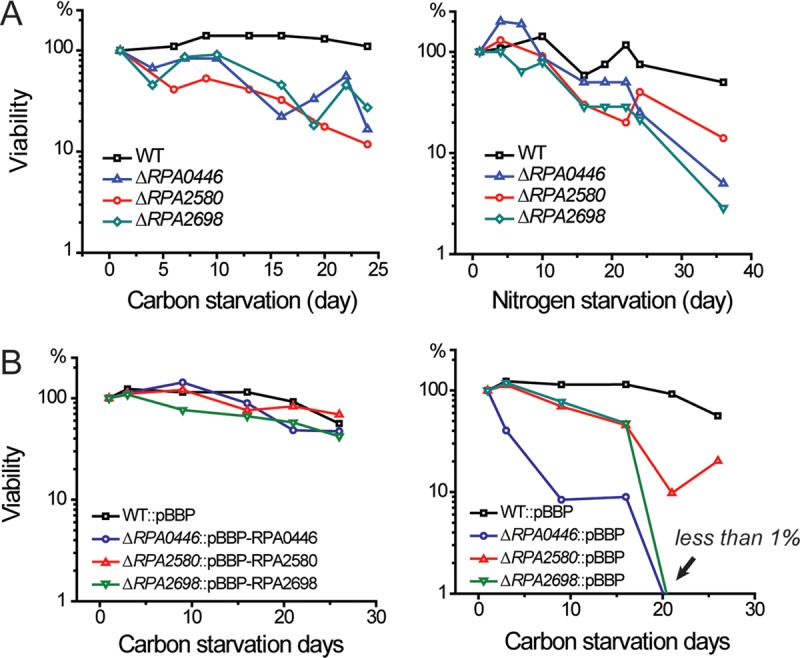
Longevity phenotypes of *R. palustris* wild-type and Δ*RPA0446*, Δ*RPA0446*, and Δ*RPA2698* deletion mutant strains. Cells were subjected to growth arrest due to either carbon starvation or nitrogen starvation according to conditions described in the text, and cell viabilities were determined by measuring CFU. In both cases, argon was present as the headspace gas in culture tubes. (A) We show one representative set of survival curves for mutants. These experiments were repeated three times, and the full sets of survival curves are shown in Fig. S2. (B) Each mutant longevity phenotype was complemented by expressing the corresponding gene in *trans*. The empty vector control is also shown.

In any Tn-seq experiment, it is important to validate mutants that appear to be important for fitness under the condition being interrogated. Toward this end, we made in-frame deletions in the five candidate genes listed in Table 2. The deletion mutants were subjected to growth arrest due to (i) acetate depletion in a medium that included ammonium as the nitrogen source and (ii) nitrogen depletion imposed by replacing the N_2_ gas in the headspace of culture tubes with argon. In both situations, growth was arrested when cells reached an OD_660_ of between 0.45 and 0.55 and longevity was assessed by recovery of colony-forming units (CFU). Three out of the five strains that we tested, the Δ*RPA0446*, Δ*RPA2580*, and Δ*RPA2698* deletion mutants, showed compromised longevities after growth arrest due to either carbon starvation or nitrogen starvation (Fig. 4A; see Fig. S2 in the supplemental material). These genes, which are homologous to genes that have been studied in *E. coli*, are annotated as encoding a 16S rRNA endonuclease, a protein l-isoaspartate *O*-methyltransferase, and a RAS-like GTPase involved in ribosome assembly (34–38). The Δ*RPA2115* mutant, defective in cyanate hydratase (39), decreased slightly in viability after growth arrest due to carbon source limitation but maintained wild-type levels of viability under nitrogen starvation conditions. The fifth strain, the Δ*RPA2183* mutant, which lacks a gene annotated to encode a DsbA thiolsulfide oxidoreductase (40), did not have a longevity phenotype (Fig. S2). The Δ*RPA0446* and Δ*RPA2580* mutants had wild-type growth rates on acetate when either ammonium or nitrogen gas was supplied as a nitrogen source. The Δ*RPA2698* strain had a slightly lower growth rate than the wild type when ammonia was the nitrogen source (Table 3).

10.1128/mBio.01726-17.2 FIG S2  Longevity phenotypes of *R. palustris* mutants compared to wild type. The results of all longevity trials are shown. The same set of survival curves for wild-type cells is shown in each panel. Download FIG S2, DOCX file, 0.4 MB. Copyright © 2017 Pechter et al. 2017 Pechter et al. This content is distributed under the terms of the Creative Commons Attribution 4.0 International license.

**TABLE 2:** Validation of candidate longevity genes

Gene mutated	Annotation	Longevity phenotype
*RPA0446*	Possible rRNA maturation endonuclease	Defective in survival after growth arrest
*RPA2115*	Cyanate hydratase (CynS)	Slight defect in survival after carbon depletion-induced growth arrest
*RPA2183*	DsbA thiol disulfide oxidoreductases	Wild-type longevity
*RPA2580*	Protein l-isoaspartate *O*-methyltransferase	Defective in survival after growth arrest
*RPA2698*	Possible GTP-binding protein involved in rRNA maturation	Defective in survival after growth arrest

**TABLE 3:** Generation times of longevity mutants

Strain	Generation time (h) for:	
	Nitrogen-fixing growth^a^	Non-nitrogen-fixing growth^b^
WT	10.7 ± 1.0	6.4 ± 0.2
Δ*RPA0446* mutant	9.8 ± 0.7	7.2 ± 0.9
Δ*RPA2580* mutant	12.1 ± 1.5	6.3 ± 0.1
Δ*RPA2698* mutant	10.0 ± 1.0	8.1 ± 0.3

a Nitrogen-fixing growth in NFM plus 20 mM sodium acetate.

b Non-nitrogen-fixing growth in PM plus 10 mM sodium acetate.

The longevity defects of the Δ*RPA0446*, Δ*RPA2580*, and Δ*RPA2698* strains were complemented by expressing the corresponding genes in *trans* (Fig. 4B). Gentamicin was included to maintain the complementing vector. Interestingly, Δ*RPA0446* and Δ*RPA2698* mutants carrying empty vector had a more severe longevity defect than mutant cells that did not carry the vector. Whereas 10 to 50% of mutant cells were viable after more than 20 days of carbon starvation in light (Fig. 4A; Fig. S2), less than 1% of either strain survived the same period of growth arrest when carrying an empty vector (Fig. 4B). One possibility is the mutant strains were hypersensitive to residual gentamicin present in the medium that was not inactivated by the gentamicin resistance gene carried on the plasmid.

## DISCUSSION

Basta et al. have described genes essential for viability of *P. aeruginosa* during energy-limited growth arrest (41). As might be expected, a subset of these genes are important for energy generation. This article also summarizes work suggesting that energy limitation is a common cause of growth arrest for heterotrophic bacteria in natural environments. Some bacteria can circumvent this problem of energy-limited growth by generating energy from light. It has been suggested that marine bacteria use proteorhodopsin to capture light energy, providing a survival advantage in nutrient-scarce oceans (42–44). Indeed, expression of proteorhodopsin in the heterologous host *Shewanella oneidensis* helped it to survive significantly longer during stationary phase (45). We have demonstrated here and previously that *R. palustris* remains viable for weeks when not growing as long it is incubated in light, a condition that drives ATP synthesis (Fig. 1). This allowed us to apply the approach of Tn-seq in *R. palustris* to identify longevity genes. The annotations of these genes suggest that cells are metabolically active when in a growth-arrested state. Under these conditions, *R. palustris* is likely required to maintain protein synthesis and to repair and maintain chromosome integrity. The inventory of longevity genes that we compiled also suggests that it is important for cells to maintain transport functions—possibly to recapture nutrients that are leaked from growth-arrested cells that have degraded ribosomes and proteins that are no longer needed to support rapid growth.

In carrying out Tn-seq experiments to identify longevity genes, we made the unexpected finding that *hoxJ* mutants that were in our Tn pool dominated the growth-arrested cultures by day 30 of carbon starvation, presumably because such mutants could grow on CO_2_ and H_2_ that accumulated in the growth-arrested cultures due to acetate oxidation and nitrogen fixation (Fig. S1). Because of this, we analyzed our data for genes that had reduced insertion fitness in cultures at 3, 7, and 14 days following growth arrest, before the *hoxJ* mutants started taking over. To increase the stringency of our analysis, we validated selected longevity genes by testing their longevity phenotypes in cultures subjected to growth arrest by two methods: (i) acetate depletion in a medium that included ammonia rather than N_2_ gas as a nitrogen source and (ii) nitrogen depletion in a medium that had acetate in excess.

Global changes in transcription occurred in *R. palustris* in the first 12 h after growth arrest. We hypothesize that these changes helped cells adapt to growth arrest based on our findings that 24 h of exposure to light following growth arrest delayed the bulk cell death that occurred when cells were subsequently deprived of energy by incubation in the dark. In view of this, we were surprised to find that there was very little overlap between genes that were increased in expression following growth arrest and the set of genes that we identified as longevity genes by Tn-seq. There are several possible interpretations for this result. One is that genes that are more highly expressed following growth arrest may provide cells with an advantage, but the advantage is not sufficiently large for us to pick up the genes as essential for longevity using the rather stringent criteria of the Tn-seq screen. Also, the Tn-seq approach cannot identify genes that are functionally redundant with another gene. *R. palustris* encodes, for example, 16 extracytoplasmic function (ECF) RNA polymerase sigma factors, many of which were increased in expression following growth arrest, but none of these showed up in our longevity gene set (Fig. 3). This could be due to overlapping functions of these auxiliary sigma factors. The stationary-phase sigma factor *rpoS*, identified as a longevity gene in *P. aeruginosa* (41) and in *E. coli* (13, 46), is not present in *R. palustris* or other alphaproteobacteria.

We expect that our longevity gene set will vary depending on the growth-limiting nutrient used to establish growth arrest. Some transporter genes likely fall into this category. At the same time, some *R. palustris* genes—particularly those involved in core cellular functions—are likely to be important for longevity regardless of the specific condition used. In our analysis, longevity genes involved in DNA repair, tRNA modification, and the fidelity of protein synthesis were critically important after, but not before, growth arrest. This suggests that there is less margin for error for some mutants to remain viable when not growing compared to when they are actively growing. Longevity genes in this category tend to be conserved among bacteria.

The verified longevity gene *RPA2580* is annotated as coding for a protein l-isoaspartyl *O*-methyltransferase (PIMT). PIMTs are found in most bacteria, but they have mainly been studied in animals and plants, where they function to repair aged proteins that can become nonfunctional due to the spontaneous deamidation and isomerization of aspartates and asparagines (36, 47–49). These modifications, which are one of the most common types of protein damage, result in the accumulation of isoapartate residues, which cause protein damage by generating kinks in polypeptide chains. One study in *E. coli* showed that mutants lacking the *pcm* gene encoding PIMT had decreased survival when challenged with an environmental stress, such as heat, salt, or hydrogen peroxide (50). Although it is tempting to conclude that RPA2580 functions in the repair of damaged proteins, this *R. palustris* protein is missing critical residues shown to be important for interaction with PIMT substrates. Thus, it will be important to verify the activity of this gene product in *in vitro* assays. Of the other two longevity genes that we verified, *RPA0446* is a homologue of *E. coli ybeY*, coding for an RNase that influences the maturation of rRNAs and plays key roles in ribosome quality control (37, 38). The other gene, *RPA2698*, is annotated as a Ras-like small GTPase gene. Its product’s homologue, Era, is essential for *E. coli* growth and provides a checkpoint for cell cycle regulation (51) and ribosome assembly (34, 35). Depletion of Era from *E. coli* leads to morphological abnormalities (52, 53). It is interesting that the *R. palustris* Δ*RPA0446* and Δ*RPA2698* mutants had no obvious growth defects, when their homologues are essential for or strongly affect *E. coli* growth (38, 53). This could be due to differences in the host species, or it may be that the *R. palustris* proteins have different functions.

The results of these studies encourage us to believe that *R. palustris* is a good model microbe for use in identifying genes that are important for the longevity of diverse bacterial species. The most logical approach is to focus now on verifying and characterizing genes that are conserved in other bacteria and on testing the effects of mutations in homologous genes on the viabilities of other bacteria that are in a state of growth arrest. More than one-third of the *R. palustris* longevity gene set is comprised of genes of unknown functions. Many of these have homologues in other bacteria, providing interesting territory to explore in the future.

## MATERIALS AND METHODS

### Bacterial strains, growth, and incubation conditions.

*R. palustris* CGA009, a *hupV* mutant lacking uptake hydrogenase activity, was used as the wild-type strain for this study (33). Cells were grown under anaerobic conditions in light as described previously (12). Two previously described defined media were used: PM, which contains ammonium sulfate (10 mM) as a nitrogen source, and a nitrogen-free medium, NFM, which is identical to PM but lacks ammonium sulfate (54). Both media had N_2_ gas as a headspace in sealed cultures, except where noted. For the Tn-seq screen, 50-ml volumes of medium were placed into stoppered 170-ml serum bottles and the cultures were stirred slowly. In-frame deletion mutants of strain CGA009 were constructed as described previously (54). For genetic complementation experiments, *R. palustris* genes were cloned into plasmid pBBPgdh, with their native ribosome binding site maintained (55). The plasmids were mobilized into *R. palustris* strain CGA009 by conjugation with to *E. coli* S17-1 as described previously (54). Media were supplemented with kanamycin (100 μg/ml), gentamicin (100 μg/ml), and/or chloramphenicol (10 μg/ml) as indicated.

For carbon starvation experiments, *R. palustris* was grown anaerobically in light with constant stirring in either NFM or PM, as indicated, supplemented with 10 mM sodium acetate. The exhaustion of acetate (OD_660_ of ~0.55) induced carbon starvation; this point was designated 0 h, or *t*0. For nitrogen starvation experiments, *R. palustris* was grown anaerobically in light in NFM supplemented with 20 mM sodium acetate. At mid-log phase (OD_660_ of ~0.45), the headspace was exchanged with argon gas to induce nitrogen starvation; this point was designated 0 h poststarvation, or *t*0. For Tn-seq experiments, it was necessary to remove dead cells through an outgrowth step, as DNA sequencing techniques cannot differentiate between DNA from living versus dead cells. Samples of growth-arrested cells were plated on CA agar (PM supplemented with 02% Casamino Acids and 0.5% yeast extract) containing sodium acetate (20 mM) medium, and plates were incubated for 5 to 7 generations under aerobic conditions in ambient light. Nutrient agar (Difco) or CA agar plates were used to determine viable CFU, as indicated.

### RNA-seq analysis of carbon-starved cells.

*R. palustris* was grown under nitrogen-fixing conditions with acetate as a limiting carbon source as described above. Control samples were removed during log phase (OD_660_ of 0.25). Samples were taken 12, 50, 90, and 180 h post-growth arrest. RNA was extracted from 5-ml samples using the RNeasy minikit (Qiagen), and samples were prepared for RNA-seq using previously described methods and the *R. palustris*-specific primer set v2 (15). RNA samples were sequenced, and the standard Illumina preprocessing pipeline was used for initial default filtering (Illumina, Inc.). Filtered reads were analyzed using Xpression, an RNA-seq bioinformatics pipeline developed by our laboratory (56).

### Genetic analysis of *R. palustris* carbon starvation by Tn-seq.

The CGA009 T24 mutant library described previously (24) was grown under nitrogen-fixing carbon-limited conditions as described above; an aliquot (2 × 10^9^ CFU) of the library was used as a culture inoculum, and the medium was supplemented with kanamycin. Cultures were sampled at *t*0, and at 3, 7, 14, and 30 days post-growth arrest to monitor OD_660_ and viable CFU and to prepare Tn-seq samples. For Tn-seq samples, culture aliquots (0.5 ml) were removed and immediately plated on large (15-cm) petri plates containing CA agar supplemented with 100 µg/ml kanamycin and 10 µg/ml chloramphenicol. Plates were incubated for 3 days at 30°C under aerobic conditions with low ambient light. Cells were scraped up and suspended in 1 ml sterile PM. An aliquot (10 µl) of the cell suspension was used to determine viable CFU. The remaining cell suspension was pelleted by centrifugation and stored at −80°C. Genomic DNA was extracted and Tn-seq samples were prepared for Illumina sequencing and sequenced as described previously (24), using the Tn circle method (57). Sequencing data were analyzed using a Tn-seq bioinformatics pipeline previously described by our laboratory (24).

Reduced- and increased-fitness knockouts were determined for each starvation time point as described previously (24). Briefly, for each biological replicate, the first 5% and last 10% read counts for each gene were discarded, and the central 5 to 90% read counts per gene were totaled and normalized to gene length (where 1 kb = 1 gene), resulting in reads per kilobase (RpK) values for each gene. Log_2_ RpK values were analyzed via a histogram and fit to a normal distribution using the nonlinear least-squares fit (Prism, GraphPad Software, Inc.), and the mean and standard deviation of the distribution were calculated. For each time point, genes with log_2_ RpK values falling outside a 95% confidence interval (*P* < 0.05 by two-tailed test) and below the mean in both biological replicates were considered genes with reduced insertion fitness, while those genes satisfying the same criteria above the mean were considered genes with increased insertion fitness. Genes with values not statistically significant from the mean were considered genes with neutral insertion fitness.
