# Supplementary figures and images for: Molecular Basis of Bacterial Longevity
Source: mBio. 2017 Nov 28;8(6):e01726-17. doi: 10.1128/mBio.01726-17 (PMC5705917; doi:10.1128/mBio.01726-17)

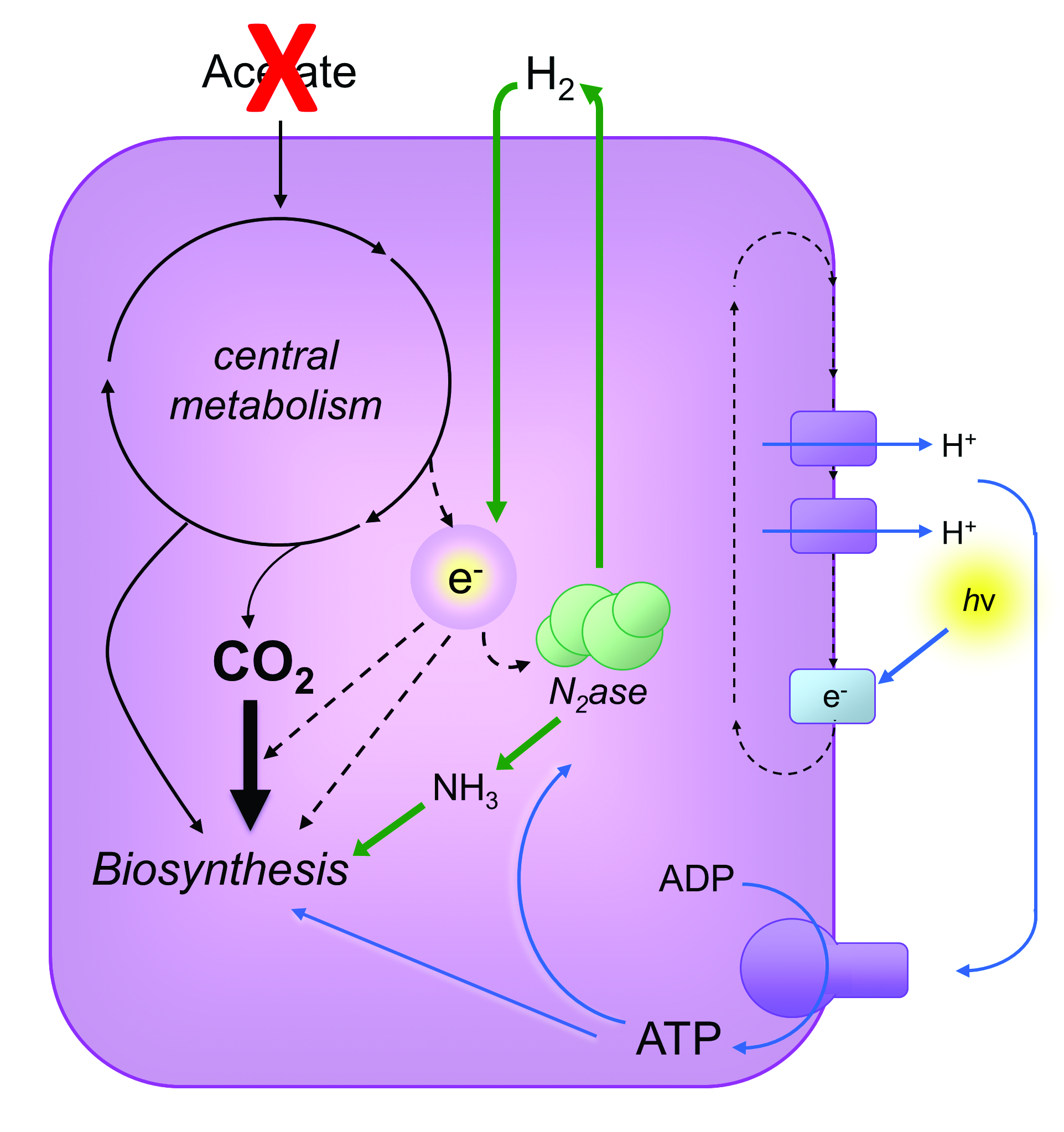

Supplement: FIG S1 [file mbo006173625sf1.tif]

Fig S2A


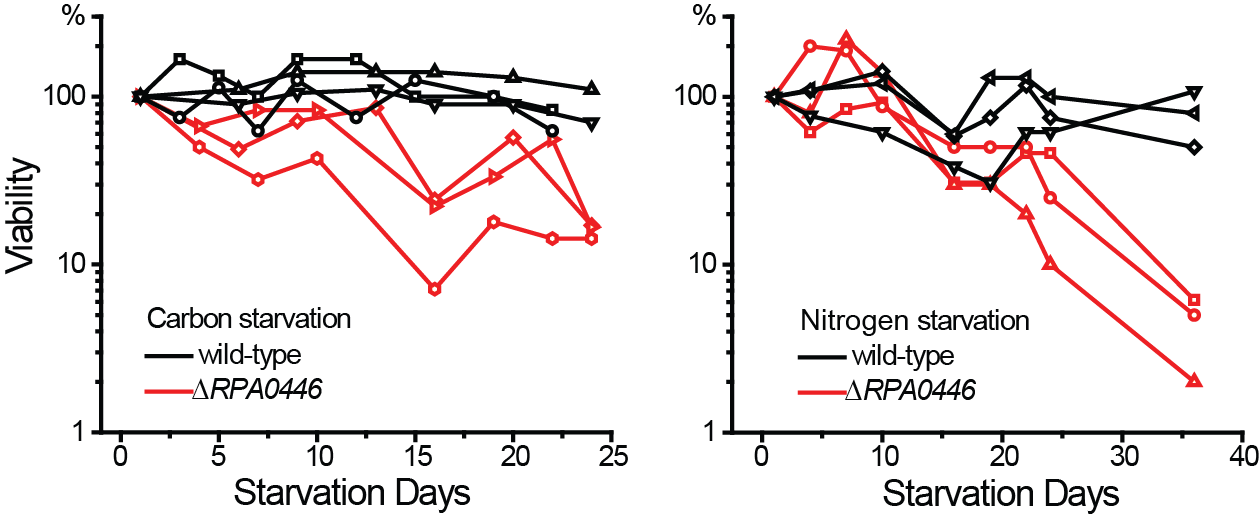


Fig. S2B


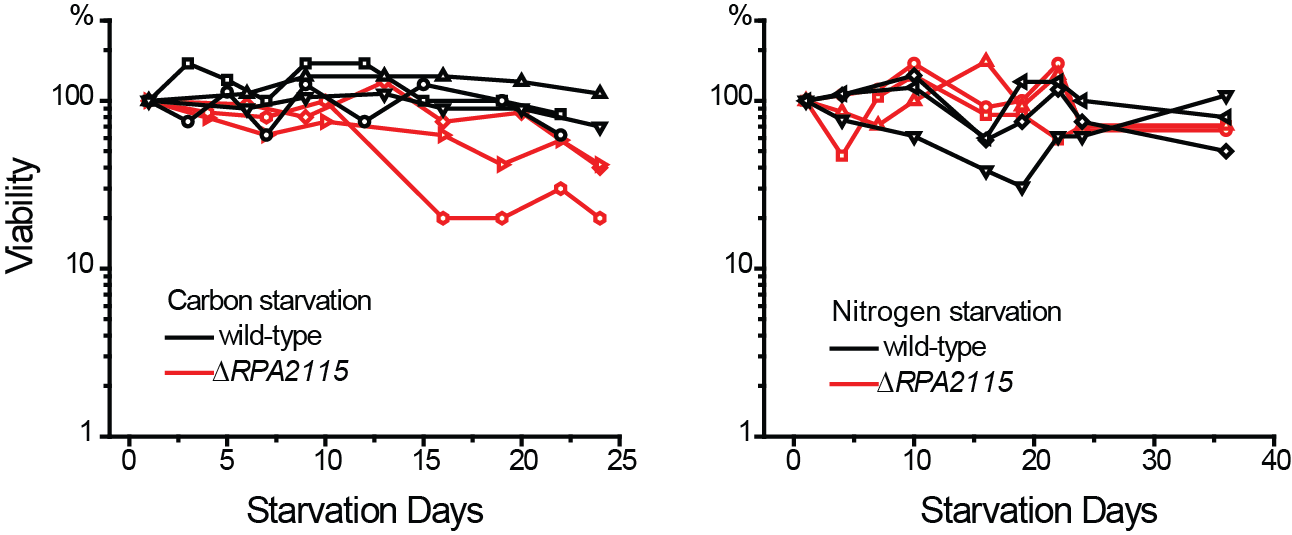


Fig. S2C


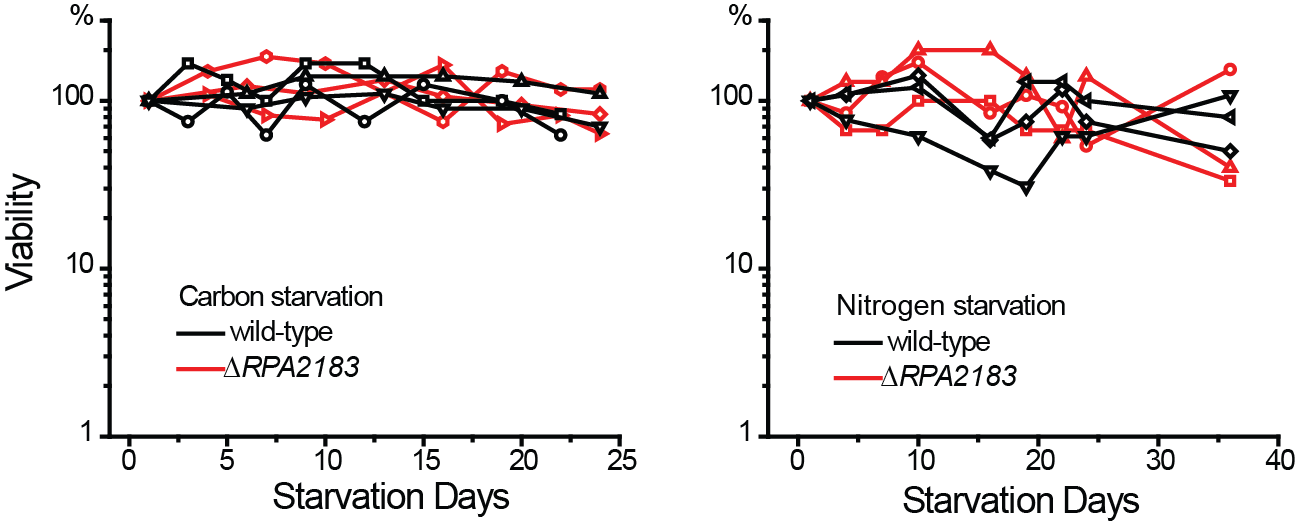


Fig S2D


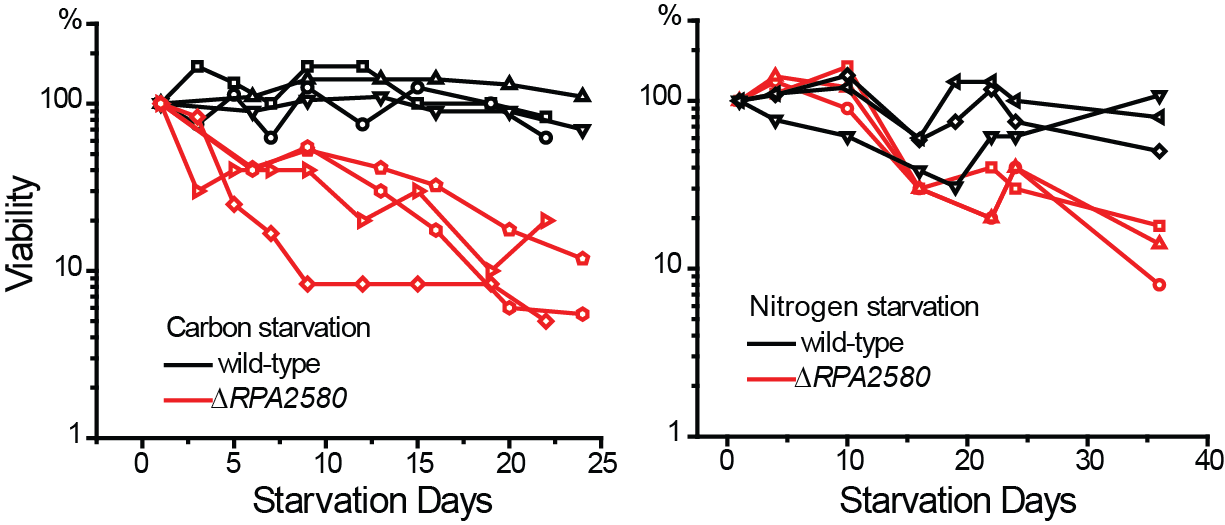


Fig S2E


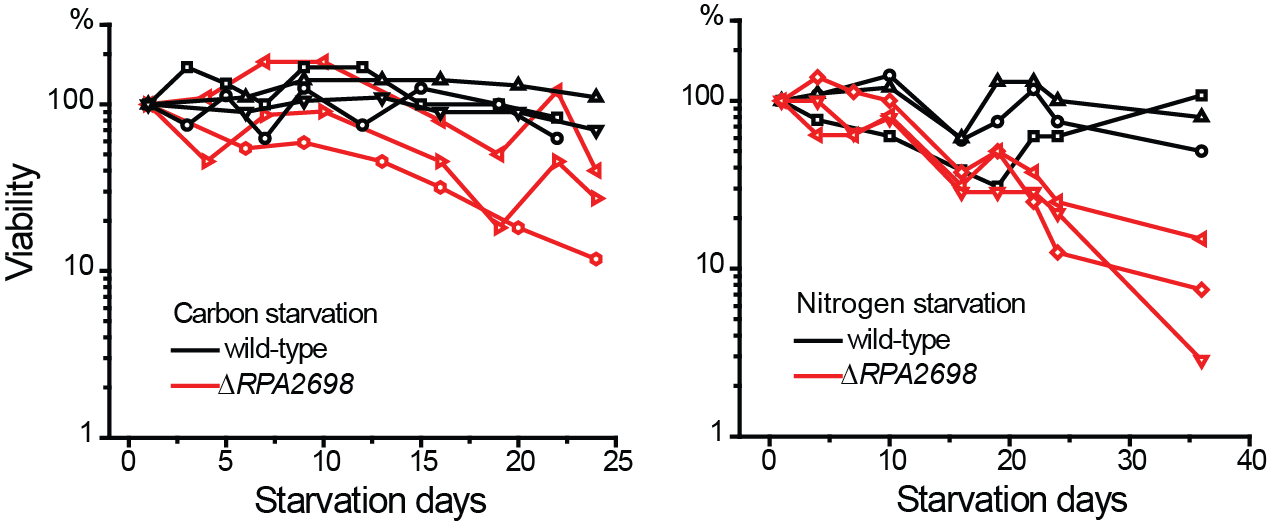

Supplement: FIG S2 [file mbo006173625sf2.docx]
